# Vaccine-induced antibody Fc-effector functions in humans immunized with a combination Ad26.RSV.preF/RSV preF protein vaccine

**DOI:** 10.1128/jvi.00771-23

**Published:** 2023-10-30

**Authors:** Yannic C. Bartsch, Deniz Cizmeci, Dansu Yuan, Nickita Mehta, Jeroen Tolboom, Els De Paepe, Roy van Heesbeen, Jerald Sadoff, Christy A. Comeaux, Esther Heijnen, Benoit Callendret, Galit Alter, Arangassery Rosemary Bastian

**Affiliations:** 1 Ragon Institute of MGH, MIT, and Harvard, Cambridge, Massachusetts, USA; 2 Department of Biological Engineering, Massachusetts Institute of Technology, Cambridge, Massachusetts, USA; 3 Janssen Vaccines & Prevention B.V., Leiden, South Holland, the Netherlands; 4 Janssen Infectious Diseases, Beerse, Belgium; Loyola University Chicago, Maywood, Illinois, USA

**Keywords:** respiratory syncytial virus, vaccines, functional antibodies, Fc-effector functions

## Abstract

An Ad26.RSV.preF/RSV preF protein combination vaccine demonstrated 80.0% vaccine efficacy for the prevention of respiratory syncytial virus (RSV)-mediated lower respiratory tract disease in a phase 2b study. In addition to neutralizing antibodies, Fc-effector functions are associated with protective immunity against RSV. Here, vaccine-induced Fc-effector functions were evaluated for the Ad26.RSV.preF/RSV preF protein vaccine in a subset of participants enrolled in a phase 1/2a study. RSV preF-specific antibody subclasses, isotypes, Fcγ receptor binding, and Fc-effector functions were evaluated on days 1 (pre-vaccination), 15, 29, and 183. Compared with Ad26.RSV.preF or RSV preF protein alone, the combination vaccine induced greater Fc-effector functions, including antibody-dependent neutrophil phagocytosis and antibody-dependent natural killer cell activation. Despite RSV pre-exposure, antibody-dependent neutrophil phagocytosis and antibody-dependent natural killer cell activation were not observed at baseline but were induced *de novo* following vaccination. Compared with the individual vaccine components, the combination vaccine induced a more polyfunctional antibody response.

**IMPORTANCE:**

Respiratory syncytial virus (RSV) can cause serious illness in older adults (i.e., those aged ≥60 years). Because options for RSV prophylaxis and treatment are limited, the prevention of RSV-mediated illness in older adults remains an important unmet medical need. Data from prior studies suggest that Fc-effector functions are important for protection against RSV infection. In this work, we show that the investigational Ad26.RSV.preF/RSV preF protein vaccine induced Fc-effector functional immune responses in adults aged ≥60 years who were enrolled in a phase 1/2a regimen selection study of Ad26.RSV.preF/RSV preF protein. These results demonstrate the breadth of the immune responses induced by the Ad26.RSV.preF/RSV preF protein vaccine.

## INTRODUCTION

Despite near-universal pre-exposure, respiratory syncytial virus (RSV) can cause severe lower respiratory tract disease (LRTD) among older adults (1), individuals with immunocompromising conditions, and adults with underlying chronic cardiac or pulmonary conditions (2 – 4). RSV is among the most common causes of acute respiratory infection (ARI), with an estimated 64 million ARIs globally each year in adults and children attributed to RSV (5). RSV is particularly burdensome to older adults; there are an estimated 177,000 hospitalizations and 14,000 deaths annually among US adults aged ≥65 years resulting from RSV infection (6). In infected older adults, RSV carries a disease burden similar to or greater than that of influenza (7, 8). Despite this substantial disease burden, RSV prophylaxis and treatment options for older adults are limited.

Infection with RSV does not confer long-lasting immunity (9). RSV reinfection occurs frequently throughout life, potentially within weeks of prior infection (10) and despite the presence of neutralizing antibodies (11). Although RSV-neutralizing antibodies are significantly correlated with protection from RSV infection in both animal models (12 – 14) and human studies (15), there is increasing evidence from both animal and human studies that CD4+ and CD8+ T cell responses (16 – 20), antibody Fc-effector functions [i.e., antibody-dependent cellular phagocytosis (ADCP), antibody-dependent cellular cytotoxicity, antibody-dependent complement deposition (ADCD), and antibody-dependent natural killer cell activation (ADNKA)], and antigen-specific immunoglobulin (Ig) isotypes (e.g., serum and mucosal IgA) and subclasses also play a critical role in protection (21, 22). Given these recent advancements in understanding the crucial aspects of immunity required to protect against RSV infection, an effective prophylactic RSV vaccine will need to induce robust, durable cellular and humoral immune responses, including neutralizing antibodies and a polyfunctional Fc-effector function profile.

Ad26.RSV.preF is a recombinant, replication-incompetent adenovirus type 26 (Ad26)-based RSV vaccine encoding a conformation-stabilized RSV pre-fusion F (preF) protein. A combination regimen consisting of Ad26.RSV.preF and a recombinant RSV preF protein has demonstrated a promising vaccine efficacy of 80.0% for the prevention of RSV-mediated LRTD in older adults (aged ≥65 years) (23). In this exploratory analysis, we used a systems serology approach to compare vaccine-induced RSV preF-specific antibody subclasses, isotypes, Fcγ receptor (FcγR) binding, and Fc-effector functions in participants receiving Ad26.RSV.preF, RSV preF protein, or the Ad26.RSV.preF/RSV preF protein combination vaccine in a phase 1/2a regimen selection study and to determine the contributions of each component to the overall vaccine-induced immune response.

## RESULTS

### Study participants and clinical procedures

Samples were analyzed from a subset of participants enrolled in a phase 1/2a study (ClinicalTrials.gov Identifier: NCT03502707) evaluating the safety and immunogenicity of Ad26.RSV.preF, RSV preF protein, and Ad26.RSV.preF/RSV preF protein combination vaccine regimens in adults aged ≥60 years. On day 1, participants received Ad26.RSV.preF [1 × 10^11^ viral particles (vp)], RSV preF protein (150 µg), the combination Ad26.RSV.preF/RSV preF protein vaccine (1 × 10^11^ vp/150 µg), or placebo. Venous blood samples were obtained pre-vaccination on day 1 and on days 15, 29, and 183 for the evaluation of RSV preF-specific antibody subclasses and isotypes, FcγR binding, and Fc-effector functions. In this exploratory analysis, changes in antibody titers and Fc-effector functions were evaluated and reported as geometric mean fold increase (GMFI) from baseline and 95% confidence intervals (CIs). Baseline immune responses are shown in Fig. S1.

### Humoral immune responses induced by Ad26.RSV.preF alone and the Ad26.RSV.preF/RSV preF protein combination vaccine

Initially, we compared antibody and Fc-effector immune responses induced by Ad26.RSV.preF alone and the Ad26.RSV.preF/RSV preF protein combination vaccine among participants in the regimen selection cohort of the phase 1/2a clinical study to determine whether the addition of RSV preF protein improved the immune responses induced by Ad26.RSV.preF in humans, as observed in previous preclinical studies (24).

**Fig 1:**
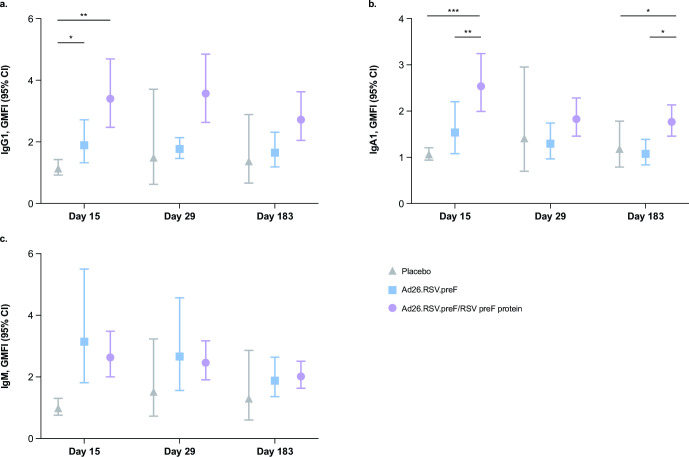
RSV preF-specific humoral immune responses induced by Ad26.RSV.preF and the Ad26.RSV.preF/RSV preF protein combination vaccine. (a) IgG1, (b) IgA1, and (c) IgM GMFIs were measured in serum samples collected on days 1 (pre-vaccination), 15, 29, and 183 from participants receiving Ad26.RSV.preF alone (*n* = 12), the Ad26.RSV.preF/RSV preF protein combination vaccine (*n* = 42), or placebo (*n* = 6). Statistical comparisons were performed by analysis of variance (Kruskal-Wallis) with a Benjamini-Hochberg correction for multiple comparisons. Error bars denote 95% CIs. *P ≤ 0.05; **P ≤ 0.01; ***P < 0.001. Ad26, adenovector type 26; CI, confidence interval; GMFI, geometric mean fold increase; Ig, immunoglobulin; preF, pre-fusion conformation-stabilized RSV F protein; RSV, respiratory syncytial virus.

Pre-existing RSV preF-specific IgG1 and IgA1 antibodies were detected at baseline, with no substantial differences between vaccination groups (Fig. 1). Both Ad26.RSV.preF alone and the Ad26.RSV.preF/RSV preF protein combination vaccine elicited robust IgG1 and IgA1 responses (Fig. 1a and b). At day 15, Ad26.RSV.preF alone increased IgG1 titers by 1.9-fold from baseline and the Ad26.RSV.preF/RSV preF protein combination vaccine increased IgG1 titers by 3.4-fold from baseline, both of which were substantially greater than placebo. IgG1 responses remained elevated from baseline up to day 183 for both active vaccine groups (GMFI, Ad26.RSV.preF alone: 1.7; Ad26.RSV.preF/RSV preF protein combination vaccine: 2.7). At day 15, the Ad26.RSV.preF/RSV preF protein combination vaccine increased IgA1 titers by 2.5-fold from baseline, which was substantially greater than both Ad26.RSV.preF alone (GMFI: 1.5) and placebo; this response was maintained up to day 183. IgM antibodies were not substantially present at baseline; both Ad26.RSV.preF alone and the Ad26.RSV.preF/RSV preF protein combination vaccine increased IgM titers from baseline to day 15 (GMFI, Ad26.RSV.preF alone: 3.1; Ad26.RSV.preF/RSV preF protein combination vaccine: 2.6), and titers remained elevated up to day 183 (Fig. 1c). GMFIs and 95% CIs for RSV preF-specific antibody responses are available in Table S1.

### Fc receptor binding and Fc-effector function induction by Ad26.RSV.preF alone and the Ad26.RSV.preF/RSV preF protein combination vaccine

**Fig 2:**
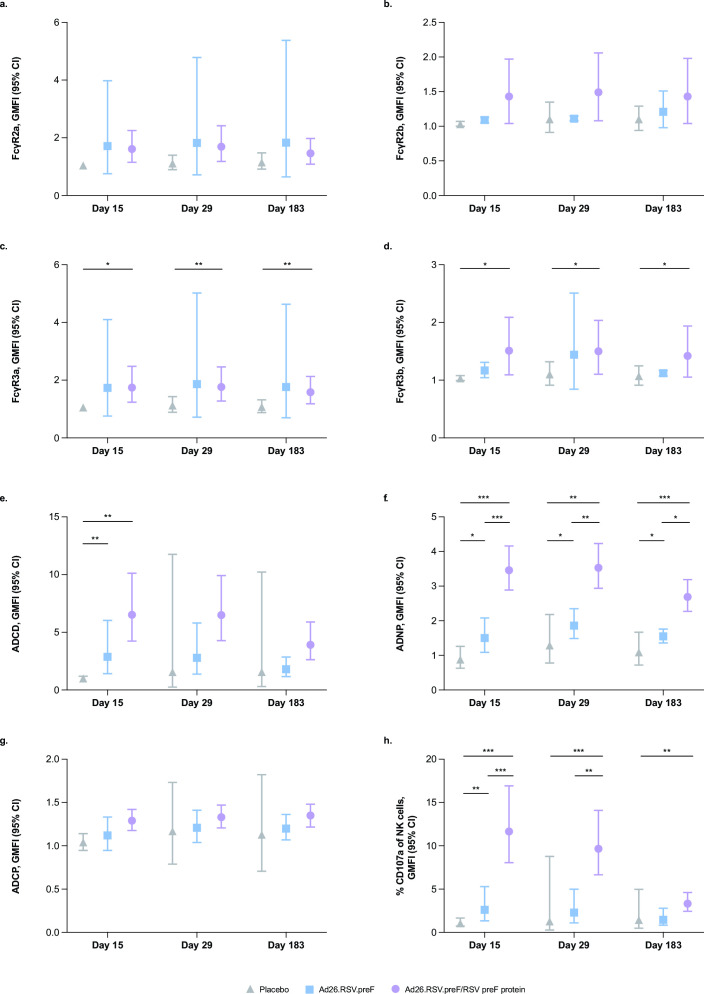
RSV preF-specific FcγR binding and Fc-effector functions induced by Ad26.RSV.preF and the Ad26.RSV.preF/RSV preF protein combination vaccine. RSV preF-specific FcγR binding and Fc-effector functions were measured in serum samples collected on days 1 (pre-vaccination), 15, 29, and 183 from participants receiving Ad26.RSV.preF alone (*n* = 12), the Ad26.RSV.preF/RSV preF protein combination vaccine (*n* = 42), or placebo (*n* = 6). (a) FcγR2a, (b) FcγR2b, (c) FcγR3a, and (d) FcγR3b binding, and induction of (e) ADCD, (f) ADNP, (g) ADCP, and (h) ADNKA, reported as GMFI from baseline. Statistical comparisons were performed by analysis of variance (Kruskal-Wallis) with a Benjamini-Hochberg correction for multiple comparisons. Error bars denote 95% CIs. *P ≤ 0.05; **P ≤ 0.01; ***P < 0.001. Ad26, adenovector type 26; ADCD, antibody-dependent complement deposition; ADCP, antibody-dependent cellular phagocytosis; ADNKA, antibody-dependent natural killer cell activation; ADNP, antibody-dependent neutrophil phagocytosis; CI, confidence interval; FcγR, Fcγ receptor; GMFI, geometric mean fold increase; NK, natural killer; preF, pre-fusion conformation-stabilized RSV F protein; RSV, respiratory syncytial virus.

Both pre-existing and vaccine-induced RSV preF-specific antibodies demonstrated binding to Fc receptors FcγR2a, FcγR2b, FcγR3a, and FcγR3b and induction of Fc-effector functions (Fig. 2). Vaccination with Ad26.RSV.preF alone or the Ad26.RSV.preF/RSV preF protein combination vaccine augmented Fc receptor binding and Fc-effector function responses. There were no substantial differences in FcγR2a or FcγR2b binding for antibodies induced by Ad26.RSV.preF, the Ad26.RSV.preF/RSV preF protein combination vaccine, or placebo (Fig. 2a and b); however, antibodies induced by the Ad26.RSV.preF/RSV preF protein combination vaccine demonstrated substantially greater FcγR3a binding (GMFI, day 15: 1.7; day 29: 1.8; day 183: 1.6; Fig. 2c) and FcγR3b binding (GMFI, day 15: 1.5; day 29: 1.5; day 183: 1.4; Fig. 2d) at all time points post-vaccination compared with placebo. GMFIs and 95% CIs for FcγR binding are available in Table S2.

Despite pre-exposure to RSV infection, substantial ADCD, antibody-dependent neutrophil phagocytosis (ADNP), and ADNKA responses were not observed at baseline, suggesting that responses measured post-vaccination were *de novo*. At day 15, ADCD was substantially increased in participants receiving either Ad26.RSV.preF alone (GMFI: 2.9) or the Ad26.RSV.preF/RSV preF protein combination vaccine (GMFI: 6.5) compared with placebo (Fig. 2e). Compared with placebo, ADNP was substantially increased in participants receiving either Ad26.RSV.preF alone (GMFI, day 15: 1.5; day 29: 1.9; day 183: 1.5) or the Ad26.RSV.preF/RSV preF protein combination vaccine (GMFI, day 15: 3.5; day 29: 3.5; day 183: 2.7) at all time points measured post-vaccination (Fig. 2f). The Ad26.RSV.preF/RSV preF protein combination vaccine induced substantially greater ADNP compared with Ad26.RSV.preF alone at all time points measured post-vaccination. Compared with placebo, vaccine-induced antibodies elicited similar ADCP responses at all time points measured (Fig. 2g). The Ad26.RSV.preF/RSV preF protein combination vaccine elicited substantially higher ADNKA compared with placebo at all time points measured post-vaccination (GMFI, day 15: 11.6; day 29: 9.7; day 183: 3.3) and substantially higher ADNKA compared with Ad26.RSV.preF alone at day 15 (GMFI: 2.6) and day 29 (GMFI: 2.3; Fig. 2h). GMFIs and 95% CIs for RSV preF-specific Fc-effector function induction are available in Table S3.

### Mechanism of vaccine-induced immune responses

Because we observed that the addition of RSV preF protein to Ad26.RSV.preF improved some antibody and Fc-effector function responses induced by Ad26.RSV.preF alone, we used a limited number of samples from the initial safety cohort of the regimen selection study to further evaluate the specific contributions of Ad26.RSV.preF and RSV preF protein to vaccine-induced immune responses. Baseline-corrected RSV preF-specific humoral immunity and Fc-effector functions were profiled on day 15 for participants receiving the Ad26.RSV.preF/RSV preF protein combination vaccine, Ad26.RSV.preF alone, RSV preF protein alone, or placebo.

**Fig 3:**
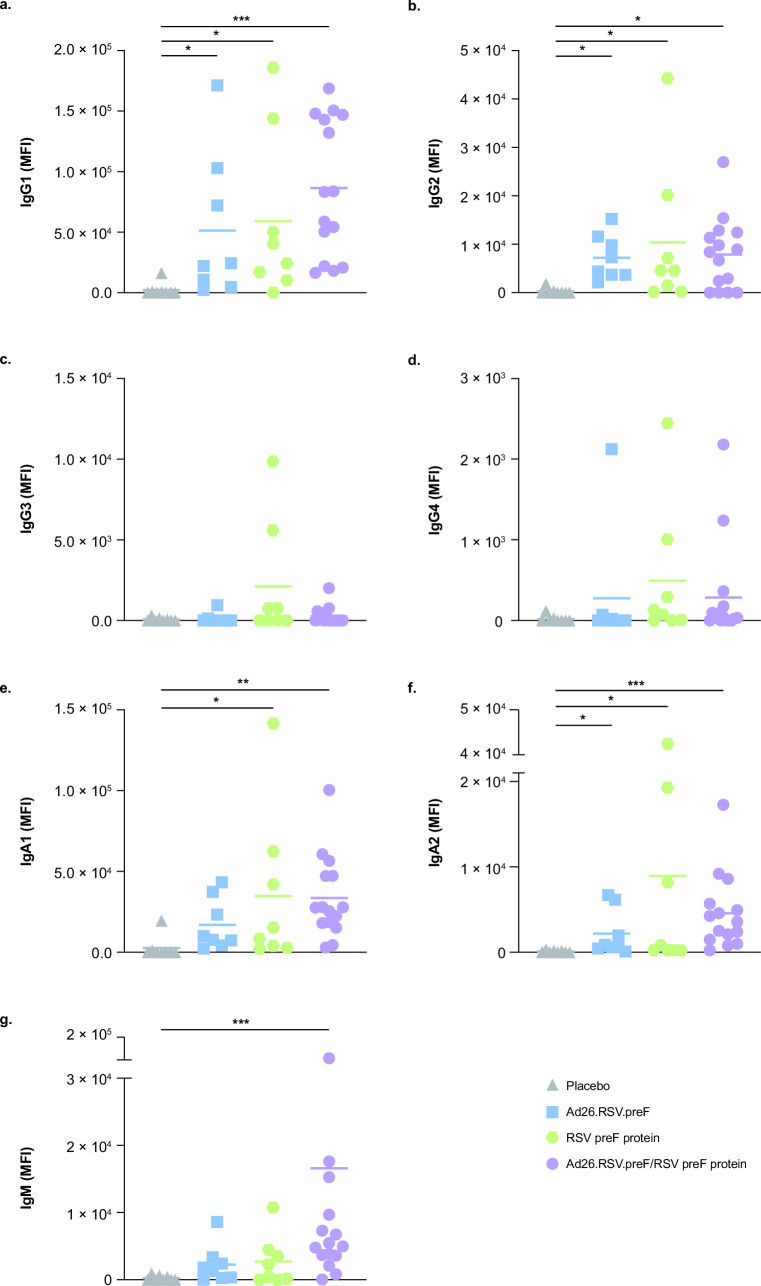
RSV preF-specific IgG, IgA, and IgM trajectories. RSV preF-specific (a) IgG1, (b) IgG2, (c) IgG3, (d) IgG4, (e) IgA1, (f) IgA2, and (g) IgM were measured in serum samples collected at day 15 for participants receiving the Ad26.RSV.preF/RSV preF protein combination vaccine (*n* = 15), Ad26.RSV.preF alone (*n* = 8), RSV preF protein alone (*n* = 8), or placebo (*n* = 8). Responses at day 15 were baseline corrected (day 0, prior to vaccination), and only positive values are shown. Horizontal lines denote geometric mean values in each group. Statistical comparisons were performed by non-parametric analysis of variance (Kruskal-Wallis) with Benjamini-Hochberg correction for multiple comparisons. *P ≤ 0.05; **P ≤ 0.01; ***P < 0.001. Ad26, adenovector type 26; Ig, immunoglobulin; MFI, median fluorescence intensity; preF, pre-fusion conformation-stabilized RSV F protein; RSV, respiratory syncytial virus.

Consistent with the results above, the Ad26.RSV.preF/RSV preF protein vaccine induced substantial increases in RSV preF-specific IgG1, IgG2, IgA1, IgA2, and IgM compared with placebo (Fig. 3). Compared with placebo, Ad26.RSV.preF alone substantially increased RSV preF-specific IgG1, IgG2, and IgA2, while RSV preF protein alone substantially increased RSV preF-specific IgG1, IgG2, IgA1, and IgA2 (Fig. 3a through f). Neither Ad26.RSV.preF nor RSV preF protein substantially increased RSV preF-specific IgM compared with placebo (Fig. 3g).

**Fig 4:**
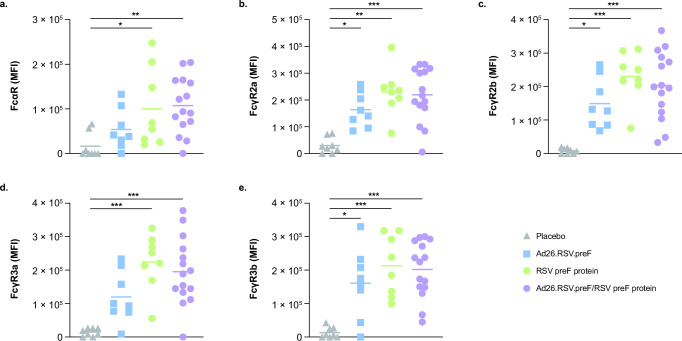
RSV preF-specific Fc receptor binding induced by Ad26.RSV.preF, RSV preF protein, and the Ad26.RSV.preF/RSV preF protein combination vaccine. RSV preF-specific (a) FcαR, (b) FcγR2a, (c) FcγR2b, (d) FcγR3a, and (e) FcγR3b binding were measured in serum samples collected at day 15 for participants receiving the Ad26.RSV.preF/RSV preF protein combination vaccine (*n* = 15), Ad26.RSV.preF alone (*n* = 8), RSV preF protein alone (*n* = 8), or placebo (*n* = 8). Horizontal lines denote geometric mean values in each group. Responses at day 15 were baseline corrected (day 0, prior to vaccination), and only positive values are shown. Statistical comparisons were performed by non-parametric analysis of variance (Kruskal-Wallis) with Benjamini-Hochberg correction for multiple comparisons. *P ≤ 0.05; **P ≤ 0.01; ***P < 0.001. Ad26, adenovector type 26; FcαR, Fcα receptor; FcγR, Fcγ receptor; MFI, median fluorescence intensity; preF, pre-fusion conformation-stabilized RSV F protein; RSV, respiratory syncytial virus.

Antibodies elicited by the Ad26.RSV.preF/RSV preF protein combination vaccine also showed substantial binding to Fc receptors, including FcαR, FcγR2a, FcγR2B, FcγR3a, and FcγR3b, compared with placebo, consistent with the results above (Fig. 4). Compared with placebo, antibodies elicited by both Ad26.RSV.preF and RSV preF protein alone also showed substantial binding to all Fc receptors evaluated, except Ad26.RSV.preF did not induce substantial binding to FcαR or FcγR3a.

**Fig 5:**
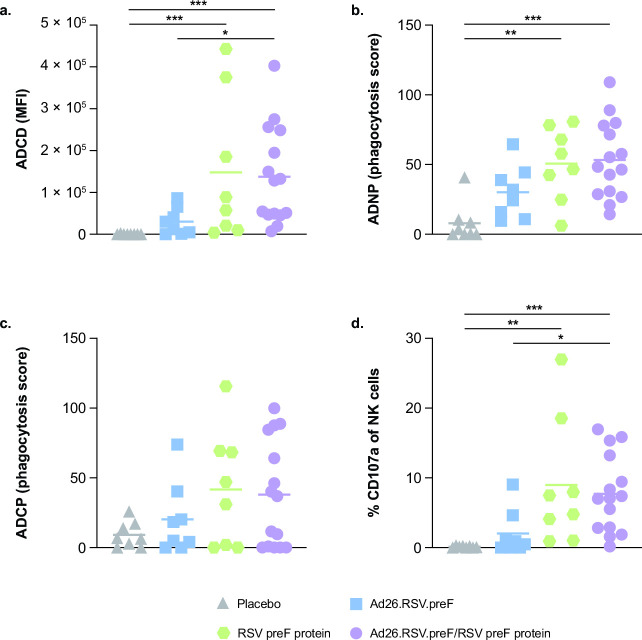
RSV preF-specific Fc-effector functions induced by Ad26.RSV.preF, RSV preF protein, and the Ad26.RSV.preF/RSV preF protein combination vaccine. RSV preF-specific (a) ADCD, (b) ADNP, (c) ADCP, and (d) ADNKA were measured in serum samples collected at day 15 for participants receiving the Ad26.RSV.preF/RSV preF protein combination vaccine (*n* = 15), Ad26.RSV.preF alone (*n* = 8), RSV preF protein alone (*n* = 8), or placebo (*n* = 8). Responses at day 15 were baseline corrected (day 0, prior to vaccination), and only positive values are shown. Horizontal lines denote geometric mean values in each group. Statistical comparisons were performed by non-parametric analysis of variance (Kruskal-Wallis) with Benjamini-Hochberg correction for multiple comparisons. *P ≤ 0.05; **P ≤ 0.01; ***P < 0.001. Ad26, adenovector type 26; ADCD, antibody-dependent complement deposition; ADCP, antibody-dependent cellular phagocytosis; ADNKA, antibody-dependent natural killer cell activation; ADNP, antibody-dependent neutrophil phagocytosis; MFI, median fluorescence intensity; NK, natural killer; preF, pre-fusion conformation-stabilized RSV F protein; RSV, respiratory syncytial virus.

The Ad26.RSV.preF/RSV preF protein combination vaccine showed substantial induction of RSV preF-specific Fc-effector functions, including ADCD, ADNP, and ADNKA, compared with placebo, consistent with the results above (Fig. 5). Compared with placebo, RSV preF protein alone substantially induced ADCD, ADNP, and ADNKA; conversely, Ad26.RSV.preF alone did not show substantial induction of any of the Fc-effector functions evaluated. The Ad26.RSV.preF/RSV preF protein combination vaccine induced substantially greater ADCD and ADNKA compared with Ad26.RSV.preF alone. A visual representation of the differential immune responses elicited by all vaccine regimens is shown in Fig. S2.

**Fig 6:**
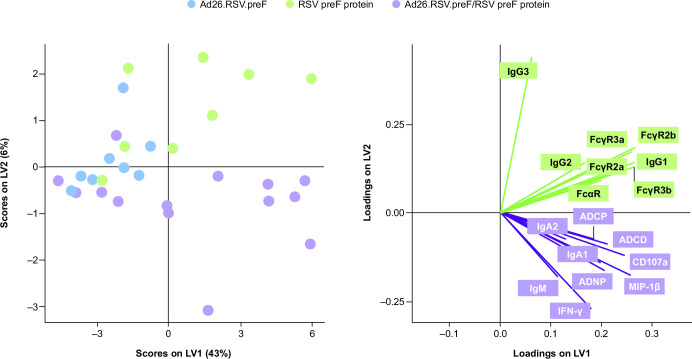
PLS-R analysis of humoral and Fc-effector responses. PLS-R analysis was used to profile humoral and Fc-effector responses for participants receiving Ad26.RSV.preF alone (*n* = 8), RSV preF protein alone (*n* = 8), or the Ad26.RSV.preF/RSV preF protein combination vaccine (*n* = 15). Ad26, adenovector type 26; ADCD, antibody-dependent complement deposition; ADCP, antibody-dependent cellular phagocytosis; ADNP, antibody-dependent neutrophil phagocytosis; FcαR, Fcα receptor; FcγR, Fcγ receptor; IFN-γ, interferon-γ; Ig, immunoglobulin; LV, latent variable; MIP, macrophage inflammatory protein; PLS-R, partial least squares regression; preF, pre-fusion conformation-stabilized RSV F protein; RSV, respiratory syncytial virus.

Partial least squares regression (PLS-R) analysis of immune responses induced by the Ad26.RSV.preF/RSV preF protein combination vaccine, RSV preF protein alone, and Ad26.RSV.preF alone showed that each regimen induced distinct immune responses (Fig. 6). While both RSV preF protein alone and the Ad26.RSV.preF/RSV preF protein combination vaccine produced high antibody titers, the Ad26.RSV.preF/RSV preF protein combination vaccine elicited higher Fc-effector functionality. Compared with participants receiving RSV preF protein alone, RSV preF-specific IgM and IgA1 were substantially increased in participants receiving the Ad26.RSV.preF/RSV preF protein combination vaccine; conversely, RSV preF-specific IgG3 and IgG4 were substantially increased in participants receiving RSV preF protein alone (Fig. S3).

## DISCUSSION

RSV vaccine development has been hindered by an incomplete understanding of the crucial aspects of the immune response required for protection from RSV infection and the lack of a universally agreed-upon correlate of protection in the RSV-experienced population. Additionally, infection with RSV does not confer long-lasting immunity, and waning or impaired immunity with age is thought to contribute to the RSV disease burden in older adults (2, 25). In addition to serum-neutralizing antibodies, recent studies suggest that mucosal (i.e., IgA) antibodies (22, 26) and antibody Fc-effector functions (21, 22) are critical to provide complete, durable protection from RSV infection, although these data are limited, and further studies are required to confirm the degree of protection provided by these immune responses. The Ad26.RSV.preF/RSV preF protein vaccine has demonstrated 80.0% vaccine efficacy for the prevention of RSV LRTD in older adults (23), and there are data from other Ad26-vectored vaccine regimens demonstrating robust induction of Fc-effector function responses in clinical trials, which may confer protection (27 – 30). Indeed, systems serology profiling of Ad26-vectored HIV and severe acute respiratory syndrome coronavirus 2 (SARS-CoV-2) vaccines demonstrated a specific immune profile enriched for Fc-effector functions, similar to the results observed in this study (29 – 32). In this context, we sought to thoroughly evaluate the humoral and antibody Fc-effector responses induced by the investigational Ad26.RSV.preF/RSV preF protein combination vaccine and determine the contributions of each component to the overall vaccine-induced humoral immune response.

In this study, all active vaccination regimens that were tested, including Ad26.RSV.preF, RSV preF protein, and the Ad26.RSV.preF/RSV preF protein combination vaccine, induced robust RSV preF-specific IgG1, IgA1, Fc receptor binding, and Fc-effector function responses; IgG1, IgA1, and Fc receptor binding were maintained up to 6 months for Ad26.RSV.preF alone and the Ad26.RSV.preF/RSV preF protein combination vaccine. The Ad26.RSV.preF/RSV preF protein combination vaccine induced substantially greater IgA1, ADNP, and ADNKA responses compared with Ad26.RSV.preF alone, suggesting important contributions to these responses by RSV preF protein. Interestingly, some RSV preF-specific Fc-effector functions (i.e., ADCD, ADNP, and ADNKA) that were not substantially present at baseline, and thus not induced or retained from prior natural infection, were induced following vaccination with the Ad26.RSV.preF/RSV preF protein combination vaccine, suggesting a *de novo* immune response from vaccination. This pattern was mirrored by the *de novo* induction of RSV preF-specific IgM, which was only observed following vaccination with the Ad26.RSV.preF/RSV preF protein combination vaccine.

Importantly, robust neutralizing antibody responses and RSV-specific IgG antibody responses may be insufficient to provide complete protection from RSV infection, as evidenced by the failure of several RSV F-specific vaccine candidates, suggesting that a more polyfunctional immune response may be necessary for protection (21). Thus, the *de novo* induction of RSV preF-specific ADCD, ADNP, and ADNKA by the Ad26.RSV.preF/RSV preF protein combination vaccine is highly promising for the protective capacity of the vaccine, as these functions may play a critical role in protection from RSV infection (21). Through ADCD, RSV-infected cells are labeled for destruction or phagocytosis by immune cells as complement factors bind to infected cells. Once complement has been deposited on their surface, infected cells may be directly destroyed by complement-dependent cytotoxicity or phagocytosed and cleared by immune cells. ADNP and ADNKA contribute directly to the removal of infected cells, as neutrophils and NK cells phagocytose and remove or directly destroy (through the release of cytotoxic granules) infected cells. Notably, recent studies have demonstrated a correlation between vaccine-induced Fc-effector functions and protection from RSV infection (in non-human primates) (33) and severe SARS-CoV-2 disease (in humans) (34). Thus, the Fc-effector functions induced by the Ad26.RSV.preF/RSV preF protein combination vaccine provide a robust antibody-mediated immune response to RSV infection that may prevent RSV-mediated disease in vaccinated individuals.

Recent work has demonstrated a correlation between increased mucosal IgA titers and protection from RSV infection (22, 26). Additionally, a recent non-clinical study evaluating vaccine-induced immune responses and correlates of protection for six different RSV vaccines in African green monkeys found that RSV preF-specific serum IgA and IgM, Fc receptor binding, ADNKA, and ADNP were significantly correlated with lower respiratory tract protection (33). Furthermore, systems serology profiling of participants receiving Ad26.RSV.preF alone in a human challenge study demonstrated that vaccine-induced antibody and Fc-effector function responses, including IgA1, FcγR2b, and ADCP, predicted protection against RSV infection (35). Thus, the robust RSV preF-specific serum IgA1 and IgM antibody and Fc-effector function (i.e., ADCD, ADNP, and ADNKA) responses elicited by the Ad26.RSV.preF/RSV preF protein combination vaccine may substantially contribute to the protective efficacy observed with this vaccine candidate in a phase 2b clinical trial (23) and add to the protection provided by other immune markers, such as neutralizing antibodies and cellular immune responses.

Data from the multivariate immune profiling analysis suggest that the addition of RSV preF protein to Ad26.RSV.preF improves the overall Fc-effector function response, in terms of both magnitude and polyfunctionality. The Ad26.RSV.preF/RSV preF protein combination vaccine is differentiated from RSV preF protein alone by its more robust RSV preF-specific IgA1 and IgM responses and the *de novo* induction of RSV preF-specific antibodies, all of which are known to be correlated with reduced infection risk (22). PLS-R analysis showed that Ad26.RSV.preF alone, RSV preF protein alone, and the Ad26.RSV.preF/RSV preF protein combination vaccine each induces distinct immune responses. RSV preF protein alone and the Ad26.RSV.preF/RSV preF protein combination vaccine induce greater immune responses evaluated quantitatively (i.e., higher antibody titers) compared with Ad26.RSV.preF alone, whereas the Ad26.RSV.preF/RSV preF protein combination vaccine induces immune responses of higher functional quality (i.e., higher virus neutralization, FcγR binding, and Fc-mediated effector function responses) compared with either component administered alone. Notably, evidence from one study of palivizumab in cotton rats suggests that improving the functional quality of immune responses increases protection against RSV infection (36). This finding is in line with previous observations with other Ad26-vectored vaccines that also induced broad Fc-effector responses, which demonstrated a correlation with protection against HIV and SARS-CoV-2 infection in non-human primates after vaccination (37, 38). While the precise mechanism is undefined, our data suggest that Ad26.RSV.preF increases the breadth of the humoral immune responses compared with RSV preF protein alone. This could occur via two possible mechanisms; Ad26.RSV.preF may be directly sensed by the immune system (e.g., by TLR9 inducing strong interferon responses), or Ad26.RSV.preF-mediated delivery of antigen-coding DNA directly to host cells where the antigen is produced and secreted may lead to altered tissue distribution and local and systemic antigen concentrations compared with RSV preF protein alone (39).

One important limitation of this study was the small sample size. Because this was an exploratory analysis of vaccine-induced immune responses, large numbers of samples were not specifically reserved for this analysis, and the number of available samples was limited. Given that limitation, no analysis to determine immune correlates of protection was possible; further studies profiling vaccine-induced protective immune responses for the Ad26.RSV.preF/RSV preF protein vaccine are ongoing.

Importantly, the Ad26.RSV.preF/RSV preF protein combination vaccine evaluated in this study demonstrated 80.0% vaccine efficacy for prevention of RSV LRTD in a phase 2b clinical trial among adults aged ≥65 years (23), possibly due to the robust induction of binding and neutralizing antibodies, cell-mediated immune responses, and polyfunctional Fc-effector responses observed herein. Further studies exploring the correlation between vaccine efficacy and Fc-effector responses are needed. Overall, these results demonstrate that the combination Ad26.RSV.preF/RSV preF protein vaccine induces robust antibody responses and a more polyfunctional antibody response compared with Ad26.RSV.preF or RSV preF protein alone.

These results demonstrate the breadth of the immune responses induced by the Ad26.RSV.preF/RSV preF protein vaccine.

## MATERIALS AND METHODS

### Study participants and clinical procedures

Samples used in this exploratory analysis were obtained from a randomized, double-blind, phase 1/2a clinical trial (ClinicalTrials.gov Identifier: NCT03502707). The clinical trial was designed and overseen by the sponsor (Janssen Vaccines & Prevention B.V.) and was conducted in accordance with the Declaration of Helsinki and principles of Good Clinical Practice, and all participants provided written informed consent prior to participation. The secondary use of samples in this study was approved by the Mass General Brigham Healthcare Institutional Review Board. Participants aged ≥60 years were randomized to receive Ad26.RSV.preF, RSV preF protein, the Ad26.RSV.preF/RSV preF protein combination vaccine, or placebo. Serum samples were collected on day 1 (pre-vaccination) and on days 15, 29, and 183.

Participants received Ad26.RSV.preF (1 × 10^11^ vp; *n* = 24), RSV preF protein (150 µg; *n* = 8), the combination Ad26.RSV.preF/RSV preF protein vaccine (1 × 10^11^ vp/150 µg; *n* = 42), or placebo (*n* = 24). Samples from a subset of these participants were used for this analysis; sample numbers are shown in figure legends for all figures.

### Antigens and biotinylation

RSV preF antigen biotinylated at the C-terminus was provided by Janssen Vaccines & Prevention B.V. (Leiden, South Holland, the Netherlands).

### Antibody isotype and Fc receptor binding

Antigen-specific antibody isotype and subclass titers and FcγR binding profiles were evaluated using a custom multiplex Luminex assay (Luminex Corp, Austin, TX, USA), as previously described (40). Briefly, biotinylated RSV preF antigen was coupled to Luminex beads (Luminex Corp) with streptavidin (Jackson ImmunoResearch Inc., West Grove, PA, USA). Coupled beads were incubated with diluted serum samples, washed, and stained using diluted (1:100) phycoerythrin (PE)-conjugated secondary antibodies (SouthernBiotech, Birmingham, AL, USA) for IgG1 (clone: Hp6001), IgG2 (clone: 31-7-4), IgG3 (clone: HP6050), IgG4 (clone: HP6025), IgM (clone: SA-DA4), IgA1 (clone: B3506B4), or IgA2 (clone: A9604D2). For FCγR binding, a biotinylated PE-streptavidin-coupled recombinant human FCγR protein (Agilent Technologies, Santa Clara, CA, USA) was used as the secondary probe. After 1 hour of incubation, samples were washed, and relative antigen-specific antibody levels were quantified using an iQue analyzer (IntelliCyt, Albuquerque, NM, USA). All antibody levels and FcγR binding are reported as median fluorescence intensity (MFI).

### Antibody-dependent complement deposition

ADCD assays were performed as described previously (41). Briefly, biotinylated RSV preF antigen was coupled to FluoSphere NeutrAvidin beads (Thermo Fisher, Waltham, MA, USA) and incubated with 10 µL of diluted (1:250) serum samples or RSV preF IgG1 monoclonal antibody (Janssen) for 2 hours at 37°C to form immune complexes. Non-specific antibodies were removed by washing, and immune complexes were incubated with guinea pig complement (Cedarlane Laboratories, Burlington, Canada) in GVB++ buffer (Boston BioProducts, Inc., Milford, MA, USA) for 20 minutes at 37°C; complement reaction was stopped by addition of EDTA (15 mM in phosphate-buffered saline). Complement factor C3 deposited on beads was stained with anti-guinea pig C3-FITC antibody (MP Biomedicals, Irvine, CA, USA) and quantified using an iQue analyzer (IntelliCyt). ADCD was reported as MFI.

### Antibody-dependent neutrophil phagocytosis

ADNP was evaluated using a phagocytosis score, as previously described (42). Briefly, biotinylated RSV preF antigen was coupled to FluoSphere NeutrAvidin beads (Thermo Fisher) and incubated with 50 µL of diluted (1:1,000) serum samples for 2 hours at 37°C to form immune complexes. Whole-blood samples were obtained from healthy donors and lysed with ammonium-chloride-potassium buffer to isolate primary human neutrophils. Neutrophils were incubated with washed immune complexes for 1 hour at 37°C, stained with diluted (1:100) Pacific Blue conjugated anti-CD66b antibody (BioLegend, San Diego, CA, USA; clone: G10F5), fixed with 4% paraformaldehyde solution, and analyzed using an iQue analyzer (IntelliCyt).

### Antibody-dependent cellular phagocytosis

ADCP was evaluated using a phagocytosis assay with THP-1 cells, as described previously (43). Briefly, biotinylated RSV preF antigen was coupled to FluoSphere NeutrAvidin beads (Thermo Fisher) and incubated with 50 µL of diluted (1:5,000) serum samples for 2 hours at 37°C to form immune complexes. THP-1 monocytes (ATCC, Manassas, VA, USA) were added to the beads and incubated for 16 hours at 37°C. Samples were fixed with 4% paraformaldehyde solution and analyzed on an iQue analyzer (IntelliCyt). ADCP was reported as a phagocytosis score.

### Antibody-dependent natural killer cell activation

MaxiSorp enzyme-linked immunosorbent assay (ELISA) plates (Thermo Fisher) were coated with RSV preF antigen for 2 hours at room temperature and blocked with 5% bovine serum albumin (Sigma-Aldrich, St. Louis, MO, USA). Diluted (1:500) serum samples (100 µL) were added to the wells and incubated overnight at 4°C. Natural killer (NK) cells were isolated from buffy coats from healthy donors using the RosetteSep Human NK Cell Enrichment Cocktail (STEMCELL Technologies, Cambridge, MA, USA) and stimulated with recombinant human interleukin-15 (1 ng/mL; STEMCELL Technologies) at 37°C overnight. NK cells were added to the washed ELISA plate and incubated together with anti-human CD107a (BD Biosciences, Franklin Lake, NJ, USA; clone: H4A3; PE-Cy5; 1:40 dilution), brefeldin A (Sigma-Aldrich), and monensin (BD Biosciences) for 5 hours at 37°C. Cells were surface stained for CD56 (BD Biosciences; clone: B159; PE-Cy7; 1:200 dilution), CD16 (BD Biosciences; clone: 3G8; APC-Cy7; 1:200 dilution), and CD3 (BD Biosciences; clone: UCHT1; Pacific Blue; 1:800 dilution). Cells were fixed and permeabilized using the FIX & PERM™ Cell Permeabilization Kit (Thermo Fisher) and stained for intracellular macrophage inflammatory protein-1β (MIP-1β; BD Biosciences; clone: D21-1351; PE; 1:50 dilution) and interferon-γ (IFN-γ; BD Biosciences; clone: B27; FITC; 1:17 dilution). NK cells were defined as CD3^–^/CD16^+^/CD56^+^, and frequencies of degranulated (CD107a^+^), MIP-1β^+^, and IFN-γ^+^ NK cells were quantified using an iQue analyzer.

### Virus neutralization

RSV-specific neutralizing antibodies were assessed in a virus neutralization assay using A549 CCL-185 cells (ATCC), firefly luciferase-expressing RSV A2 virus, and serially diluted serum samples, as previously described (44).

### Statistical analysis

Data analysis was performed using GraphPad Prism (v9.2.0) and RStudio (v1.3 and R v4.0). Prior to analysis, all data were normalized using z-scoring. Statistical comparisons between groups were performed using Mann-Whitney *U* tests followed by Benjamini-Hochberg (BH) correction or by non-parametric analysis of variance (Kruskal-Wallis) with BH correction for multiple comparisons with an α of 0.05 in GraphPad Prism. Regression models were built using the R package “stats” (45). Correlations between immune response features at day 15 were performed using the Spearman method with BH-adjusted *P* values.

Multivariate classification models were built to discriminate humoral profiles between treatment groups on day 15. Feature selection was performed using a least absolute shrinkage and selection operator (LASSO). Classification and visualization were performed using PLS-R, and model accuracy was assessed using 10-fold cross-validation. In each run, samples were stratified into training and test subsets; for each test fold, LASSO-based feature selection was performed on logistic regression using the nine subsets designated as the training set for that fold. LASSO was repeated 100 times; features selected ≥90 times out of 100 were identified as selected features. PLS-R was applied to the training set using the selected features, and prediction accuracy was recorded. Selected features were ordered according to variable importance in the projection score, and the first two latent variables of the PLS-R model were used to visualize the samples. These analyses were performed using the R package “ropls” version 1.20.0 (46) and “glmnet” version 4.0.2 (47).

## Data Availability

The data sharing policy of Janssen Pharmaceutical Companies of Johnson & Johnson is available at https://www.janssen.com/clinical-trials/transparency. The data supporting the findings of this study may be obtained from the authors upon reasonable request.

## SUPPLEMENTAL MATERIAL

Supplemental material jvi.00771-23-s0001.docx Fig. S1 to S3 and Tables S1 to S3.

ASM does not own the copyrights to Supplemental Material that may be linked to, or accessed through, an article. The authors have granted ASM a non-exclusive, world-wide license to publish the Supplemental Material files. Please contact the corresponding author directly for reuse.
