# Supplementary material for: Vaccine-induced antibody Fc-effector functions in humans immunized with a combination Ad26.RSV.preF/RSV preF protein vaccine
Source: J Virol. 2023 Oct 30;97(11):e00771-23. doi: 10.1128/jvi.00771-23 (PMC10688327; doi:10.1128/jvi.00771-23)
Supplement: Supplemental material — Fig. S1 to S3 and Tables S1 to S3. [file jvi.00771-23-s0001.docx]

**Supplementary Information**

**Supplementary Fig 1. RSV preF-specific Fc-effector functions, IgG, IgA, IgM, and FcγR binding at baseline (Day 1) and Day 15.**

RSV preF–specific (**a**) ADNP, (**b**) ADCD, (**c**) ADCP, (**d**) ADNKA, (**e**) IgG1, (**f**) IgG2, (**g**) IgG3 (**h**) IgG4, (**i**) IgA1, (**j**) IgA2, (**k**) IgM, (**l**) FcαR, (**m**) FcγR2a, (**n**) FcγR2b, (**o**) FcγR3a, and (**p**) FcγR3b binding was measured in serum samples collected at Day 1 and Day 15 for participants receiving the Ad26.RSV.preF/RSV preF protein combination vaccine (n = 15), Ad26.RSV.preF alone (n = 8), RSV preF protein alone (n = 8), or placebo (n = 8). Horizontal lines denote geometric mean values in each group. Data are not baseline corrected.

Ad26, adenovector type 26; ADCD, antibody-dependent complement deposition; ADCP, antibody-dependent cellular phagocytosis; ADNKA, antibody-dependent natural killer cell activation; ADNP, antibody-dependent neutrophil phagocytosis; FcαR, Fcα receptor; FcγR, Fcγ receptor; Ig, immunoglobulin; MFI, median fluorescence intensity; preF, pre-fusion conformation stabilized RSV F protein; RSV, respiratory syncytial virus.


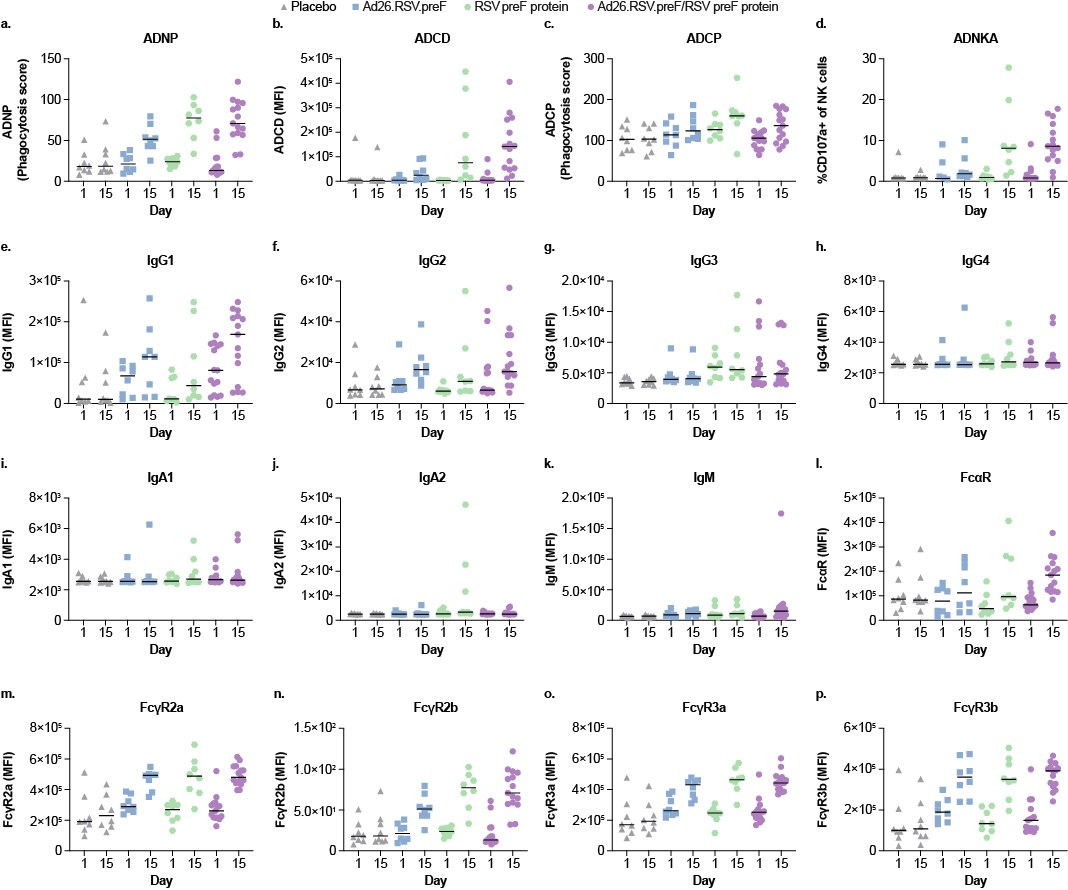


**Supplementary Fig. 2 RSV preF–specific humoral and Fc-effector response profiles.** Flower plots were generated to depict RSV preF–specific humoral and Fc-effector response profiles for participants receiving the Ad26.RSV.preF/RSV preF protein combination vaccine (n = 15), Ad26.RSV.preF alone (n = 8), RSV preF protein alone (n = 8), or placebo (n = 8), summarized by flower plots; the length of each petal represents the mean of the z-scored value for the respective color-coded feature.

Ad26, adenovector type 26; ADCD, antibody-dependent complement deposition; ADCP, antibody-dependent cellular phagocytosis; ADNP, antibody-dependent neutrophil phagocytosis; FcγR, Fcγ receptor; IFN-γ, interferon-γ; Ig, immunoglobulin; MIP, macrophage inflammatory protein; preF, pre-fusion conformation stabilized RSV F protein; RSV, respiratory syncytial virus; VNT, virus neutralizing titers.


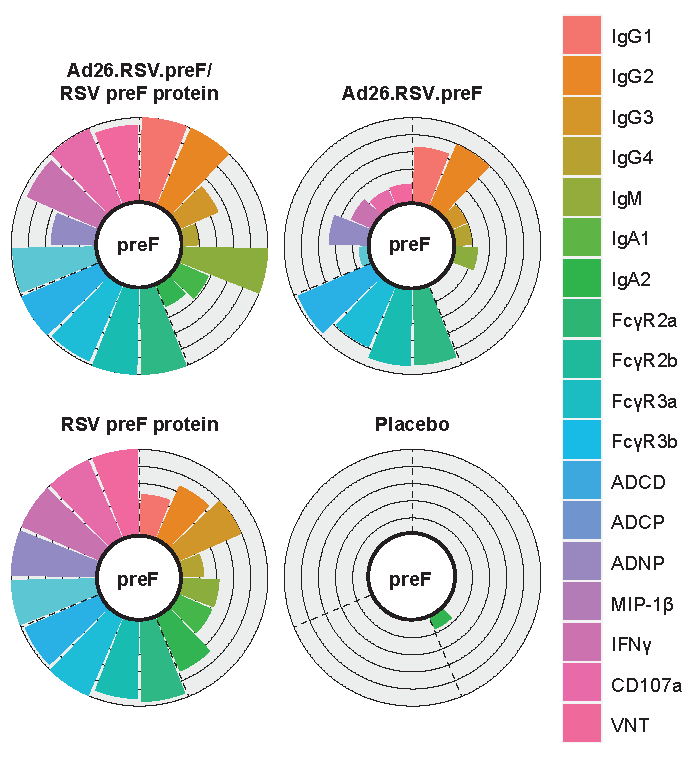


**Supplementary Fig. 3 Multivariate analysis of immune response profiles** **for RSV preF protein and Ad26.RSV.preF/RSV preF protein.** PLS-R analysis was used to compare immune response profiles of RSV preF protein alone (n = 8) with the Ad26.RSV.preF/RSV preF protein combination vaccine (n = 15).

Ad26, adenovector type 26; FcαR, Fcα receptor; FcγR, Fcγ receptor; LV, latent variable; Ig, immunoglobulin; preF, pre-fusion conformation stabilized RSV F protein; postF, post-fusion RSV F protein; RSV, respiratory syncytial virus.


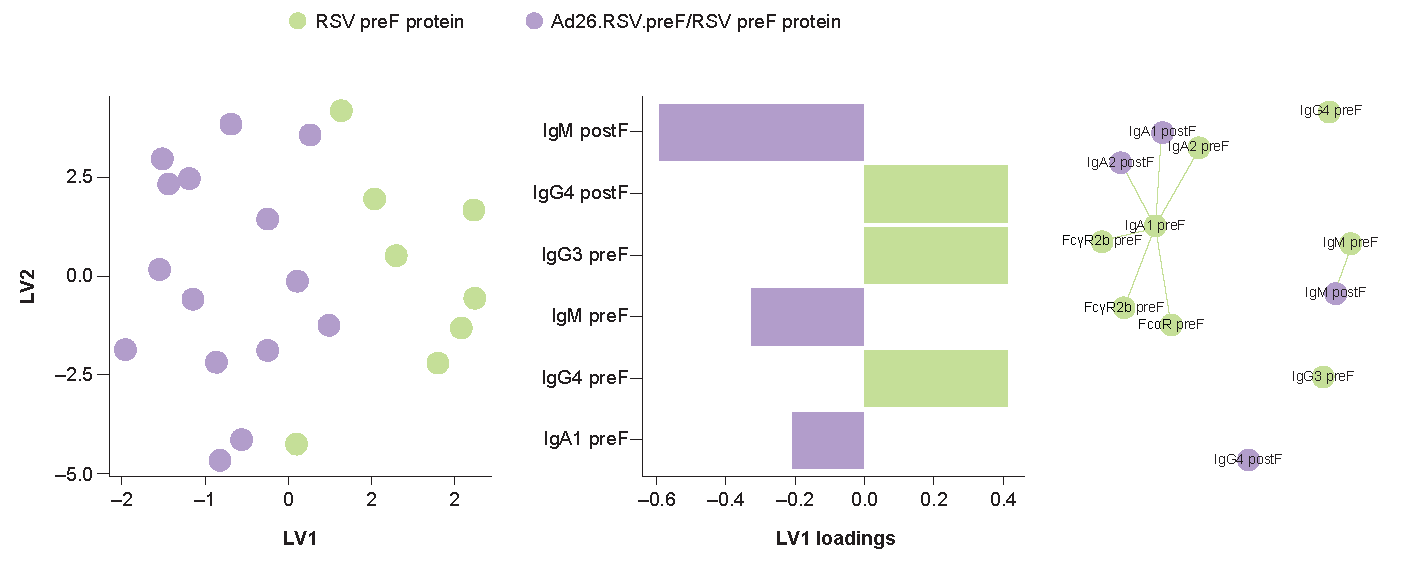


**Supplementary Table 1. GMFI (95% CI) from baseline in RSV preF–specific antibody responses at Days 15, 29, and 183.**

|  | **Ad26.RSV.preF**  **(n = 12)** | **Ad26.RSV.preF/RSV preF protein**  **(n = 42)** | **Placebo**  **(n = 6)** |
| --- | --- | --- | --- |
|  |  |  |  |
| **IgG1** |  |  |  |
| Day 15 | 1.9 (1.3, 2.7) | 3.4 (2.5, 4.7) | 1.1 (0.9, 1.4) |
| Day 29 | 1.8 (1.4, 2.2) | 3.6 (2.6, 4.9) | 1.5 (0.6, 3.7) |
| Day 183 | 1.7 (1.2, 2.3) | 2.7 (2.0, 3.6) | 1.4 (0.6, 2.9) |
| **IgG2** |  |  |  |
| Day 15 | 1.3 (1.0, 1.6) | 1.4 (1.0, 2.0) | 1.0 (0.8, 1.2) |
| Day 29 | 1.4 (1.1, 1.9) | 1.7 (1.3, 2.4) | 1.3 (0.7, 2.5) |
| Day 183 | 1.6 (1.2, 2.0) | 2.0 (1.5, 2,6) | 1.4 (0.8, 2.5) |
| **IgG3** |  |  |  |
| Day 15 | 2.1 (0.9, 4.7) | 1.9 (1.4, 2.6) | 1.0 (0.9, 1.2) |
| Day 29 | 1.7 (0.9, 3.5) | 1.7 (1.3, 2.3) | 1.2 (0.8, 1.8) |
| Day 183 | 1.1 (0.9, 1.3) | 1.2 (1.0, 1.4) | 1.1 (0.8, 1.3) |
| **IgG4** |  |  |  |
| Day 15 | 1.1 (1.0, 1.2) | 1.0 (0.9, 1.1) | 1.0 (0.9, 1.1) |
| Day 29 | 1.2 (1.0, 1.4) | 1.1 (1.0, 1.3) | 1.2 (0.9, 1.6) |
| Day 183 | 1.2 (1.0, 1.4) | 1.3 (1.1, 1.5) | 1.2 (0.8, 1.7) |
| **IgA1** |  |  |  |
| Day 15 | 1.5 (1.1, 2.2) | 2.5 (2.0, 3.3) | 1.1 (0.9, 1.2) |
| Day 29 | 1.3 (1.0, 1.7) | 1.8 (1.4, 2.3) | 1.4 (0.7, 3.0) |
| Day 183 | 1.1 (0.8, 1.4) | 1.8 (1.4, 2.1) | 1.2 (0.8, 1.8) |
| **IgA2** |  |  |  |
| Day 15 | 1.9 (1.3, 2.8) | 3.6 (2.5, 5.3) | 1.0 (0.9, 1.1) |
| Day 29 | 1.5 (1.1, 2.1) | 2.0 (1.4, 2.8) | 1.7 (0.6, 5.0) |
| Day 183 | 1.1 (0.9, 1.5) | 1.8 (1.4, 2.3) | 1.3 (0.8, 2.0) |
| **IgM** |  |  |  |
| Day 15 | 3.1 (1.8, 5.5) | 2.6 (2.0, 3.5) | 1.0 (0.7, 1.3) |
| Day 29 | 2.6 (1.5, 4.6) | 2.4 (1.9, 3.2) | 1.5 (0.7, 3.2) |
| Day 183 | 1.9 (1.3, 2.6) | 2.0 (1.6, 2.5) | 1.3 (0.6, 2.9) |

Ad26, adenovirus type 26; CI, confidence interval; GMFI, geometric mean fold increase; Ig, immunoglobulin; preF, pre-fusion conformation RSV F protein; RSV, respiratory syncytial virus.

**Supplementary Table 2. GMFI (95% CI) from baseline in RSV preF–specific FcγR binding at Days 15, 29, and 183.**

|  | **Ad26.RSV.preF**  **(n = 12)** | **Ad26.RSV.preF/RSV preF protein**  **(n = 42)** | **Placebo**  **(n = 6)** |
| --- | --- | --- | --- |
|  |  |  |  |
| **FcγR2a** |  |  |  |
| Day 15 | 1.7 (0.7, 4.0) | 1.6 (1.1, 2.3) | 1.0 (1.0, 1.1) |
| Day 29 | 1.8 (0.7, 4.8) | 1.7 (1.2, 2.4) | 1.1 (0.9, 1.4) |
| Day 183 | 1.8 (0.6, 5.4) | 1.5 (1.1, 2.0) | 1.2 (0.9, 1.5) |
| **FcγR2b** |  |  |  |
| Day 15 | 1.1 (1.0, 1.1) | 1.4 (1.0, 2.0) | 1.0 (1.0, 1.1) |
| Day 29 | 1.1 (1.1, 1.2) | 1.5 (1.1, 2.1) | 1.1 (0.9, 1.4) |
| Day 183 | 1.2 (1.0, 1.5) | 1.4 (1.0, 2.0) | 1.1 (0.9, 1.3) |
| **FcγR3a** |  |  |  |
| Day 15 | 1.7 (0.7, 4.1) | 1.7 (1.2, 2.5) | 1.0 (1.0, 1.1) |
| Day 29 | 1.9 (0.7, 5.0) | 1.8 (1.2, 2.5) | 1.1 (0.9, 1.5) |
| Day 183 | 1.8 (0.7, 4.7) | 1.6 (1.2, 2.2) | 1.1 (0.9, 1.3) |
| **FcγR3b** |  |  |  |
| Day 15 | 1.2 (1.0, 1.3) | 1.5 (1.1, 2.1) | 1.0 (1.0, 1.1) |
| Day 29 | 1.4 (0.8, 2.5) | 1.5 (1.1, 2.1) | 1.1 (0.9, 1.3) |
| Day 183 | 1.1 (1.1, 1.2) | 1.4 (1.0, 1.9) | 1.1 (0.9, 1.3) |

Ad26, adenovirus type 26; CI, confidence interval; FcγR, Fcγ receptor; GMFI, geometric mean fold increase; Ig, immunoglobulin; preF, pre-fusion conformation RSV F protein; RSV, respiratory syncytial virus.

**Supplementary Table 3. GMFI (95% CI) from baseline in RSV preF–specific Fc-effector functions at Days 15, 29, and 183.**

|  | **Ad26.RSV.preF**  **(n = 12)** | **Ad26.RSV.preF/RSV preF protein**  **(n = 42)** | **Placebo**  **(n = 6)** |
| --- | --- | --- | --- |
|  |  |  |  |
| **ADCD** |  |  |  |
| Day 15 | 2.9 (1.4, 6.1) | 6.5 (4.2, 10.2) | 1.0 (0.8, 1.2) |
| Day 29 | 2.8 (1.3, 5.9) | 6.5 (4.2, 10.0) | 1.6 (0.2, 11.8) |
| Day 183 | 1.8 (1.1, 2.9) | 3.9 (2.6, 6.0) | 1.6 (0.2, 10.3) |
| **ADNP** |  |  |  |
| Day 15 | 1.5 (1.1, 2.1) | 3.5 (2.9, 4.2) | 0.9 (0.6, 1.3) |
| Day 29 | 1.9 (1.5, 2.4) | 3.5 (2.9, 4.3) | 1.3 (0.8, 2.2) |
| Day 183 | 1.5 (1.3, 1.8) | 2.7 (2.3, 3.2) | 1.1 (0.7, 1.7) |
| **ADCP** |  |  |  |
| Day 15 | 1.1 (0.9, 1.3) | 1.3 (1.2, 1.4) | 1.0 (0.9, 1.2) |
| Day 29 | 1.2 (1.0, 1.4) | 1.3 (1.2, 1.5) | 1.2 (0.8, 1.7) |
| Day 183 | 1.2 (1.1, 1.4) | 1.3 (1.2, 1.5) | 1.1 (0.7, 1.8) |
| **ADNKA** |  |  |  |
| Day 15 | 2.6 (1.3, 5.4) | 11.6 (8.0, 17.0) | 1.1 (0.6, 1.7) |
| Day 29 | 2.3 (1.0, 5.1) | 9.7 (6.6, 14.2) | 1.3 (0.2, 8.9) |
| Day 183 | 1.5 (0.7, 2.9) | 3.3 (2.4, 4.7) | 1.4 (0.4, 5.0) |

Ad26, adenovirus type 26; ADCD, antibody-dependent complement deposition; ADCP, antibody-dependent cellular phagocytosis; ADNP, antibody-dependent neutrophil phagocytosis; ADNKA, antibody-dependent natural killer cell activation; CI, confidence interval; GMFI, geometric mean fold increase; preF, pre-fusion conformation RSV F protein; RSV, respiratory syncytial virus.
